# Deconvoluting the Biological Roles of Vitamin D-Binding Protein During Pregnancy: A Both Clinical and Theoretical Challenge

**DOI:** 10.3389/fendo.2018.00259

**Published:** 2018-05-23

**Authors:** Spyridon N. Karras, Theocharis Koufakis, Hana Fakhoury, Kalliopi Kotsa

**Affiliations:** ^1^Division of Endocrinology and Metabolism, First Department of Internal Medicine, Medical School, Aristotle University of Thessaloniki, AHEPA University Hospital, Thessaloniki, Greece; ^2^Department of Biochemistry and Molecular Biology, College of Medicine, AlFaisal University, Riyadh, Saudi Arabia

**Keywords:** vitamin D-binding protein, 25-hydroxyvitamin D, Gc-globulin, pregnancy, clinical outcomes, polymorphisms

## Abstract

The teleological purpose of an ongoing pregnancy is to fulfill its fundamental role of a successful, uncomplicated delivery, in conjunction with an optimal intrauterine environment for the developing fetus. Vitamin D metabolism is adapted to meet both these demands during pregnancy; first by stimulation of calcium absorption for adequate intrauterine bone mineral accrual of the fetus, and second, by enhancing systemic and local maternal tolerance to paternal and fetal alloantigens. Vitamin D-binding protein (VDBP) is one of the key biomolecules that optimize vitamin D homeostasis and also contributes as an immune regulator for a healthy, ongoing pregnancy. In this regard, recent results indicate that dysregulation of VDBP equilibrium could be a risk factor for adverse fetal, maternal, and neonatal outcomes, including preeclampsia, preterm birth, and gestational diabetes. Moreover, it has been hypothesized to be also implicated in the interpretation of vitamin D status in the pregnant state. The aim of this review is to assess available literature regarding the association of VDBP with clinical outcomes during pregnancy, as a potential biomarker for future clinical practice, with a discourse on current knowledge gaps and future research agenda.

## Introduction

The vitamin D-binding protein (VDBP), also known as group-specific component of serum (Gc-globulin), is a member of the albumin, α-fetoprotein, and α-albumin/afamin gene family and the major plasma carrier protein of vitamin D and its metabolites (1, 2). Vitamin D sterols are important for preserving normal serum calcium levels and electrolyte homeostasis. In addition to its specific sterol-binding capacity, VDBP has been shown to be involved in a plethora of other essential biological functions, including actin scavenging to fatty acid transport and macrophage activation and chemotaxis (2).

The teleological purpose of an ongoing pregnancy is to fulfill its fundamental role of a successful, uncomplicated delivery, in conjunction with an optimal intrauterine environment for the developing fetus. Vitamin D is adapted to meet both these demands during pregnancy; first by stimulation of calcium absorption for adequate intrauterine bone mineral accrual of the fetus, and second, by enhancing systemic and local maternal tolerance to paternal and fetal alloantigens (1–3). In this context, it is believed that VDBP is one of the key biomolecules that optimize vitamin D homeostasis and also contributes as an immune regulator for a healthy, ongoing pregnancy. VDBP concentrations are increased in the pregnant state; however, the functional significance of this fact has not, so far, fully been clarified (3). There are emerging theories, that in clinical terms and under certain conditions, biodynamics of VDBP compound could reflect the health status of an ongoing pregnancy, as well as being predictors of neonatal birth parameters or adverse outcomes (2). From an analytical aspect, VDBP could interfere with available assays and confound interpretation of maternal and neonatal vitamin D status.

The aim of this review is to assess available literature regarding the association of VDBP with clinical outcomes during pregnancy, as a potential biomarker for future clinical practice, with a discourse on current knowledge gaps and future research agenda.

## Overview of VDBP Biodynamics

### Non-Pregnant State

In humans, vitamin D_3_ (cholecalciferol) is naturally obtained through sunlight in the UVB range of 290–315 nm, through a membrane enhanced thermal-dependent isomerization reaction, which results in 7-dehydrocholesterol conversion into vitamin D_3_ (4). Alternatively, vitamin D, either as D_2_ or D_3_, can enter the body from its absorption in the intestine. In either case, D_2_ or D_3_ then diffuse into the circulation through the capillary bed and reversibly bound to the vitamin D-binding (globulin) protein (VDBP) (5). VDBP is a 58 kDa glycosylated α-globulin that carries the lipophilic vitamin D in the plasma until it reaches target tissues (6). It is composed of 458 amino acid residues in length and folds into a disulfide-bonded, triple-domain structure. The latter is further divided into two repeated, homologous domains of 186 acids (domains I and II) and a shorter domain of 86 residues at the C-terminus (domain III) (7). It is considered as the principal transporter of vitamin D molecules. Liver is the main organ where VDBP is synthesized, whereas it is also expressed in kidney, gonads and fat tissue (8).

Vitamin D_3_ undergoes its first step of activation, namely 25-hydroxylation in the liver, by the mitochondrial form of 25-hydroxylase (CYP27A1), which appears to be a bifunctional cytochrome P450 enzyme (9, 10). The product of the 25-hydroxylation step, 25-hydroxyvitamin D_3_ [25(OH)D_3_] (calcidiol), is the major circulating form of vitamin D_3_. In humans, it is present in plasma at concentrations that range between 10 and 40 ng/ml (25–125 nM) (11–13). The second step of activation occurs mainly in the kidney (14) by the cytochrome P450 enzyme, 25(OH)D-1^α^-hydroxylase (CYP27B1). This process leads to the formation of the active metabolite of vitamin D_3_, 1, 25-dihydroyxvitamin D_3_ [1, 25 (OH) 2D_3_] (calcitriol) (15, 16).

The majority of circulating 25(OH)D and 1,25-dihydroxyvitamin D is tightly bound to VDBP, with a smaller amount (10–15%) bound to albumin. Less than 1% of circulating vitamin D metabolites exists in a free, unbound form (4–6).

Apart from its function as a carrier protein, affinity for VDBP is the major parameter regulating the half-life of a vitamin D metabolite in the systemic circulation (17–19). The “free hormone hypothesis” suggests that only free steroid hormones are physiologically active, because their lipophilic ability allows them to passively diffuse across cell membranes. According to the “free hormone hypothesis,” it is only the free 25(OH)vitamin D_3_ that is taken up into the tubular epithelium, to be converted by CYP27B1 to calcitriol. Similarly, in the case of calcitriol, biological actions are mediated through passive diffusion of the free calcitriol to its cognate nuclear vitamin D receptor (VDR), which is a high-affinity ligand-activated transcription factor (13, 14).

Vitamin D-binding protein was believed to solely regulate the amount of free 25(OH)D available in the circulation (20). However, a landmark study by Nykjaer et al. (21) revealed an important transport system that affects vitamin D metabolism, being the megalin–cubilin endocytotic system. In this case, 25(OH)D/VDBP complex in the circulation is endocytosed into the proximal tubular cell *via* the apical-membrane receptor, megalin, the largest member of the LDL receptor super family (22). Megalin-mediated endocytosis of 25(OH)D/VDBP, also requires the receptor-associated protein and cubilin, a protein required for sequestering VDBP on the cell surface prior to its internalization by megalin (21). This system is a key player in the delivery of 25(OH)D to the 25-hydroxyvitamin D-1-α-hydroxylase in the kidney (21), since 25(OH)D molecules bound to VDBP taken up *via* this receptor pathway, are converted to calcitriol. The megalin–cubilin system has been also recognized in the placenta and several other tissues (22, 23). These results underline that VDBP, in addition to its carrier protein functions and regulation of free vitamin D fractions available in the circulation, presents pleiotropic actions: contributes significantly to renal and extra-renal production of calcitriol and ensures vitamin D molecule reabsorption in the kidney, by preventing urinary loss of vitamin D.

### Pregnancy

#### Systemic Circulation

A limited number of studies have determined longitudinal increase of VDBP concentrations during pregnancy (24, 25). The magnitude of the increase varies: highest concentrations reach a 40–50% increase compared to non-pregnant women, with a maximum at the beginning of the third trimester before starting to decrease at term. VDBP increase was accompanied by an increase in calcitriol concentrations in most available studies (24, 25). As expected, a negative association between free 25(OH)D and VDBP concentrations was evident, resulting in a consistent decrease of free 25(OH)D from 15 to 36 weeks of gestational age (25). On the other hand, as the affinity of calcitriol is much lower for VDBP compared to 25(OH)D, a significant increase in both total 1,25(OH)2D and VDBP levels was observed during pregnancy, while the free 1,25(OH)2D concentrations remained nearly constant (26, 27). However, whereas the increase in total 1,25(OH)2D and VDBP concentrations in the pregnant state has been repeatedly reported in different studies (24, 26), reports on the free 1,25(OH)2D are conflicting. In general, an increase in 1,25(OH)2D—being of both renal and placental origin—throughout pregnancy seems to be replicated by the majority of data (28–30). Nevertheless, observed discrepancies between different trials may actually be the result of complex interactions between calcitriol concentrations and a plethora of factors, including VDBP levels, and stimulation by prolactin, insulin-like growth factor 1, and parathyroid hormone (PTH)-related protein, while they are probably unaffected by PTH, which has been shown to decrease during pregnancy (30). Moreover, these studies used different laboratory methods of assessment of calcitriol concentrations; thus, interpretation of their results may be problematic.

Interestingly, Chun and colleagues have recently proposed a viable hypothesis considering a role for VDBP in tissue discrimination of 25(OH)D_2_ and 25(OH)D_3_ (31). Given that 25(OH)_2_ binds to VDBP with lower affinity than 25(OH)D_3_, the kidney would preferentially use the latter metabolite. Differently, cells in the immune system might profit of a greater pool of 25(OH)D_2_ for antimicrobial peptide induction (31), which is of outmost importance for enhancing systemic and local maternal tolerance to paternal and fetal alloantigens immune tolerance induction (32).

#### Placenta

Placenta has its own mechanisms regulating vitamin D metabolism. The decidua facilitates nutritional fetal–maternal exchange and serves as an endocrine tissue by secreting a plethora of biomolecules. In addition, it provides “immunological stability and tolerance” to accommodate the developing fetus. The 1^α^-hydroxylase, the 24-hydroxylase, the VDBP, and VDR have all been detected either in trophoblast cultures or in freshly obtained, placental tissue (33–37). Undoubtedly, the placenta is able to metabolize vitamin D, providing active 1,25(OH)_2_ D *in vitro*. VDBP is expressed on the cell-surface of human placental trophoblasts during normal human pregnancy (37). This observation has led to the suggestion that the rise in VDBP concentrations during pregnancy could be the result of high turnover rate of trophoblasts, which are in direct contact with maternal blood (31). VDBP has been also demonstrated to affect the expression of specific placental aminotransporters, which may be involved in the regulation of amino acid transfer to the offspring during *in utero* development (38).

**Figure 1:**
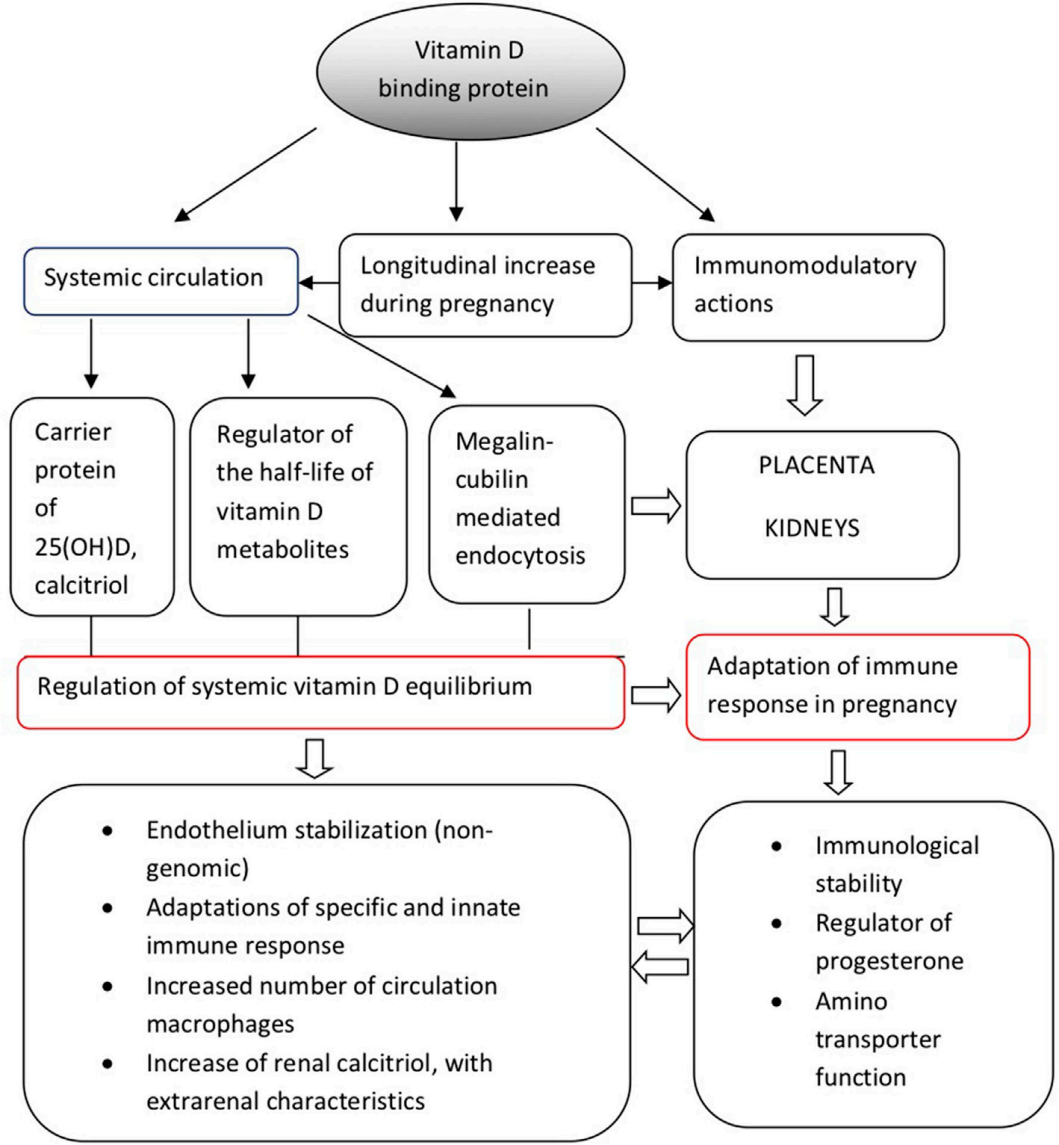
Physiological functions of vitamin D-binding protein during pregnancy.

Vitamin D-binding protein could be possibly connected with the management of large amounts of progesterone produced by the placental trophoblast during the second and third trimesters of pregnancy, which could theoretically displace vitamin D from VDBP (25, 26). Under these conditions, VDBP may additionally play the role of a major plasma progesterone transport protein at least during late gestation; however, relevant data are still scarce and this hypothesis warrants further clarification. Although these mechanisms are still under research investigation, the above observations support the multifunctional role of VDBP both as a regulator of vitamin D homeostasis and as an immunomodulator at a systemic and placental level, during pregnancy. In accordance with this hypothesis, VDBP dysregulation has been implicated in the pathogenesis of several adverse outcomes during pregnancy, which will be further discussed below. Figure 1 provides a schematic overview of the physiological functions of VDBP during pregnancy.

## VDBP as a Marker of a Healthy Ongoing Pregnancy: Clinical Implications

During the past few years, there has been increasing research effort to discover novel biomarkers that could effectively predict adverse pregnancy and fetal outcomes. In this setting, the dysfunction of the immunoregulatory biological properties of circulating VDBP during pregnancy, as well as specific VDBP polymorphisms, have been the objective of several clinical studies.

### Association With Type 1 Diabetes (T1D), Gestational Diabetes, and Adipokines

In a recent nested case-control study, concentrations of VDBP and 25(OH)D throughout pregnancy between 113 women whose offspring later developed T1D and 220 controls, were evaluated (39). VDBP and 25(OH)D significantly increased by gestational week and were lower in cases than in controls. Lower third trimester VDBP concentrations tended to be associated with higher risk of T1D in the offspring (39). Moreover, in a study among Chinese women, the risk allele-A of rs3733359 of VDBP gene was correlated with an increased risk of gestational diabetes mellitus, in the obese subgroup (40). Similar to other autoimmune disorders, the involvement of VDBP in the pathogenesis of T1D, may lay on a positive correlation between its levels and macrophage activation (41). Higher levels and frequencies of serum anti-VDBP autoantibodies were identified in patients with T1D than in healthy controls, suggesting VDBP as a possible autoantigen in T1D (42). Given that VDBP exerts immunomodulatory characteristics and contributes to the transport of vitamin D metabolites, reduced serum concentrations may be related, in a direct or indirect way, to the autoimmune functional deterioration of pancreatic β-cells in the disease.

Recent results from our group, in maternal–neonatal pairs at birth, demonstrated an independent positive correlation of VDBP with adipokines, adiponectin, and irisin, which remained significant after adjustment for multiple parameters, including weeks of gestation, maternal age, and Body Mass Index, in both mothers and neonates (not for irisin in the case of neonates) (43). Further mechanistic studies are required to elucidate whether VDBP plays a carrier or regulatory role for adiponectin and/or irisin during pregnancy and its potential effects on offspring anthropometry in late childhood and adolescence.

### VDBP and the Risk of Adverse Pregnancy Outcomes

Vitamin D-binding protein has been also implicated recently in the pathogenesis of preeclampsia. A small pilot study showed that VDBP in the first trimester of pregnancy was upregulated in women who developed early-onset preeclampsia (EOPE) compared to controls, suggesting a hypothetical VDBP utility, as a biomarker for the diagnosis of EOPE (44). These results were in accordance with a previous cohort study, which included 239 pregnant women, 107 with preeclampsia, and 132 controls, where phenotype frequency distribution of serum group-specific component (Gc) and haptoglobin (Hp) was determined (45). The results indicated a significant statistical difference in phenotype frequency distribution of the Gc-system. Gc 2-1 phenotype was expressed significantly in women with preeclampsia compared to controls, suggesting a potential utility of Gc 2-1 phenotype as a genetic marker for early preeclampsia detection (45). However, a recent study by Powe et al. showed no significance variances between first trimester VDBP concentrations between cases with preeclampsia and controls, neither association with first trimester blood pressure (46). In contrast to previous studies, Tannetta et al. showed that actin-free VDBP plasma levels tended to be lower in early onset preeclampsia compared to normal pregnancies, still not statistically significantly (47). It becomes evident that the heterogeneity of baseline 25(OH)D concentrations across trimesters of pregnancy of the populations included in these studies could contribute to this discordance. Of major interest, Behrouz et al. demonstrated that VDBP of placental origin is a target for auto-antibodies detected in sera of preeclamptic women, indicating a strong autoimmune component in the pathogenesis of the disorder (48).

Vitamin D-binding protein has been also suggested to contribute to the development of an optimal intrauterine environment for the developing fetus as well as to a successful, uncomplicated delivery. Results from the Southampton Women’s Survey (33) indicate that maternal both 25(OH)D and VDBP concentrations were positively linked to placental expression of certain genes related to placental amino acid transport. On that basis, in a recent study by Wookey et al., a significant reduction of placental VDBP concentrations in women with idiopathic fetal growth restriction, as compared to normal pregnancy controls, was demonstrated (49).

On the other hand, albumin/VDBP ratio was proven to be more efficacious than fetal fibronectin in predicting spontaneous preterm delivery in symptomatic women within 7 days (50). VDBP concentrations in cervicovaginal fluid (CVF) of pregnant women successfully predicted spontaneous labor onset within 3 days, with positive and negative predictive values of 82.8 and 95.3%, respectively (51). VDBP was estimated to be 3.9-fold higher in the CVF of asymptomatic women that subsequently presented preterm premature rupture of the fetal membranes (PROM), as compared to gestation-matched controls (52).

Potential explanations for the VDBP rise in CVF in pregnancies with high risk for preterm birth could be the increased cell death and inflammation of the fetal membranes leading to increased permeability of blood vessels and augmented VDBP deglycosylation, as an effect of the immune response (52). In addition, VDBP synthesis is known to be enhanced by pro-inflammatory cytokines, such as IL-6 (51). Given that the results of different studies regarding the VDBP concentrations in EOPE are conflicting, it has been also suggested that a potential reduction of VDBP plasma levels in EOPE, may reflect the dysfunction of the actin scavenging system, which is known to cleave extracellular actin and hinder repolymerization, inhibiting its thrombotic effects (47).

### VDBP and 25(OH)D Status During Pregnancy and Lactation

GC Single Nucleotide Polymorphism (SNPs) rs12512631 and rs7041 were determined in the peripheral blood of 356 pregnant individuals and were found to significantly interplay with the maternal and cord-blood concentrations of 25(OH)D and birth weight (49). Low 25(OH)D concentrations in the maternal and cord blood were significantly associated with decreased birth weight among infants of mothers carrying the rs12512631 “C” allele, but not in those born to mothers homozygous for the “T” allele. In addition, low 25(OH)D concentrations in cord blood were significantly linked with reduced birth weight only among infants born to mothers being carriers of the rs7041 “G” allele (53).

Vitamin D-binding protein polymorphisms have been also reported to affect vitamin D status and attained 25(OH)D concentrations after supplementation. In this regard, GC rs2282679 polymorphism was found to positively correlate with achieved 25(OH)D status, following gestational cholecalciferol supplementation (54).

There is also evidence that polymorphisms in VDBP gene may be related to 25(OH)D status during pregnancy. The minor allele for rs7041 was related to increased 25(OH)D and rs4588 was associated with decreased 25(OH)D, among pregnant women (55). Chinese pregnant women with VDBP Gc-1f and Gc-1s genotypes had higher plasma 25(OH)D concentrations compared to women with Gc-2 (56). VDBP is known to increase during pregnancy; however, this phenomenon was observed only in women with rs7041 GG or GT genotypes, while pregnant TT carriers did not manifest greater VDBP concentrations compared to TT non-pregnant controls (57).

The impact of genotype on VDBP changes during pregnancy may reflect placental vitamin D transport and thus regulate the availability of vitamin D to the mother and fetus. A different study demonstrated higher VDBP concentrations in healthy pregnant women compared to non-pregnant controls, presenting comparable vitamin D intake, suggesting that metabolic alterations, possibly involving the placenta, may occur during pregnancy that aim to increase vitamin D supply (58). In addition, genetic and ethnic variations in VDBP polymorphisms could also explain the different responses after vitamin D supplementation during pregnancy (32).

On the other hand, a recent study that explored the association between 25(OH)D and VDBP concentrations in lactating mother–neonate pairs, concluded that the high maternal and the neonatal serum VDBP concentrations may be related to falsely low vitamin D concentrations, as suggested by the normal serum calcium (Ca), phosphorus (P), magnesium (Mg), Alkaline Phosphatase (ALP), and PTH levels (59). Even when maternal and neonatal serum vitamin D concentrations were consistent with each other in terms of profound hypovitaminosis D (<10 ng/ml), this definition was not enough to establish vitamin D deficiency, without taking into account other regulatory factors of the vitamin D biological network, including Ca, P, and PTH concentrations.

### VDBP and Infertility

Vitamin D-binding protein has been also considered to be involved in the pathogenesis of idiopathic infertility. A recent pilot case-control study, including 39 infertile premenopausal women and 29 fertile controls, identified that VDBP concentrations were lower in the infertile group, compared to controls (60). In the same study, total 25(OH)D concentrations did not significantly differ between the two groups; however, free and bioavailable vitamin D concentrations were higher among the infertile women. The genotype distribution of GC rs1155563 and rs2298849 SNPs was compared between 154 women with endometriosis-associated infertility and 347 controls; still, no statistically significant differences were detected (61).

**Figure 2:**
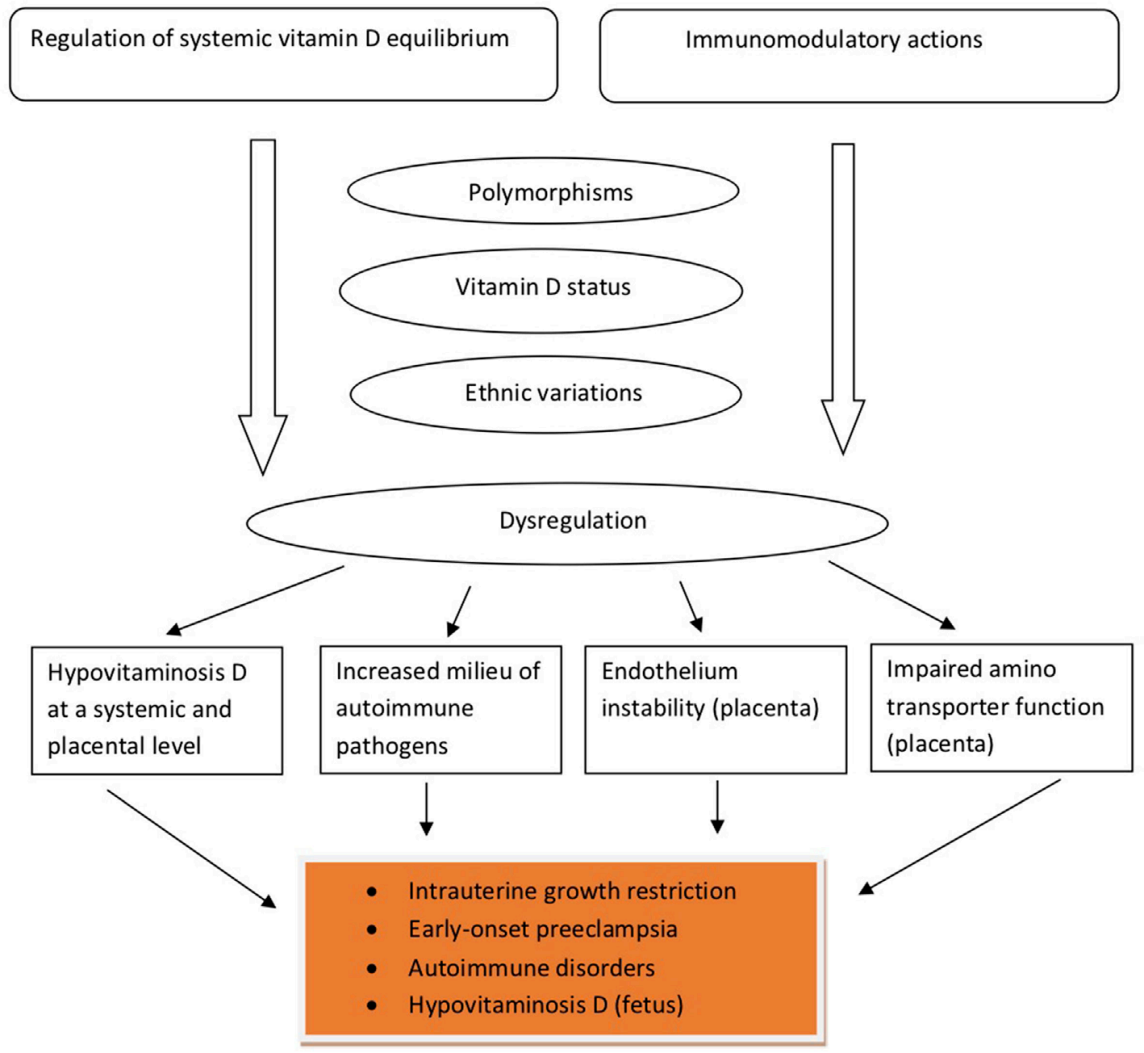
The main pathophysiologic aspects of the vitamin D-binding protein network in pregnancy.

In a cohort of 165 healthy women, aged between 26 and 75 years, it was found that postmenopausal women had higher 25(OH)D, VDBP, and estradiol concentrations than premenopausal subjects, and that estradiol was independently correlated to VDBP (62). The work by Pirani et al. demonstrated that estradiol treatment increased the uptake of labeled VDBP by hepatocytes isolated from female animals, but not from male animal cells, indicating that the estradiol effect may lay on the presence of estrogen receptors (63). Interestingly, in infertile women undergoing *in vitro* fertilization, VDBP concentrations were not found to fluctuate as estradiol changes throughout the follicular phase of the menstrual cycle (64). These findings are suggestive of the regulatory role that other factors—besides the already well known, such as age, gender and race—may play in determining VDBP concentrations. Table 1 summarizes key characteristics and findings of studies examined the relationship between VDBP and pregnancy-related clinical outcomes. Figure 2 provides a schematic overview of the main pathophysiologic aspects of the VDBP network, during pregnancy.

**Table 1:** Main characteristics and findings of key studies examined the relation between VDBP and pregnancy-related clinical outcomes.

Study ID	First author, year (reference)	Included subjects	Investigated outcome(s)	Key findings
1	Sørensen, 2016 (39)	113 pregnant women whose offspring later developed T1D/220 pregnant controls	VDBP concentrations during pregnancy and subsequent risk for T1D development in the offspring	Lower third trimester VDBP concentrations associated with higher risk of T1D in the offspring

2	Wang, 2015 (40)	692 women with GDM/802 pregnant controls	Whether Vitamin D related SNPs predispose to GDM development	rs3733359 allele-A was correlated with an increased GDM risk, in the obese subgroup

3	Kolialexi, 2017 (44)	5 pregnant women with EOPE/5 pregnant controls	Identification of potential biomarkers for EOPE	VDBP in the first trimester was upregulated to 3.38-fold in the EOPE group

4	Mekbeb, 1990 (45)	107 pregnant women with PE/132 pregnant controls	Relation between GC phenotype and PE risk	Gc 2-1 phenotype was expressed significantly in PE group compared to controls

5	Powe, 2010 (46)	39 pregnant women with PE/131 pregnant controls	Relation between first trimester VDBP levels and subsequent PE risk	No association between VDBP concentrations, subsequent PE development and first trimester BP

6	Cleal, 2015 (33)	85 pregnant women	Relation between maternal VDBP levels and placental expression of genes related to placental amino acid transport	VDBP levels were positively associated with placental expression of specific genes involved in amino acid transport

7	Wookey, 2017 (49)	Placentae from 18 pregnant women with FGR/17 gestation-matched healthy control subjects	Whether VDBP expression is altered in FGR-associated placental dysfunction	Significant reduction of placental VDBP concentrations in women with idiopathic FGR compared to controls

8	Liong, 2015 (50)	12 pregnant women with preterm delivery/129 women as validation cohort	Identification of CVF biomarkers predictive of spontaneous preterm birth in women with symptoms of preterm labor	Albumin/VDBP ratio is more efficacious than fetal fibronectin in predicting spontaneous preterm delivery in symptomatic women within 7 days

9	Liong, 2013 (52)	5 pregnant women with PROM/10 gestation-age matched controls	Identification of differentially expressed proteins in the CVF of asymptomatic women before the clinical manifestation of preterm PROM	VDBP was significantly increased (3.9-fold) in the PROM group

10	Tannetta, 2014 (47)	10 non-pregnant women/10 women with normal pregnancy/10 women with EOPE/10 women with LOPE	Investigation of the actin scavenging system in PE	Actin-free VDBP plasma levels were lower in EOPE compared to normal pregnancies, still not statistically significantly

11	Szczepańska, 2015 (61)	154 women with endometriosis-associated infertility/347 controls	Identification of genetic risk factors for endometriosis-associated infertility	Genotype distribution of GC rs1155563 and rs2298849 SNPs did not differ between patients and controls

12	Behrouz, 2013 (48)	5 human placentas from normotensive pregnant women/sera from 20 normal and 20 women with severe PE	Investigation of placental proteins as targets for auto-antibodies in PE patients	VDBP of placental origin is a target for auto-antibodies detected in sera of women with PE

13	Chun, 2017 (53)	Maternal and umbilical cord blood from 356 pregnant women and their infants	Relation between maternal GC SNPs, 25(OH)D concentrations and infant birth weight	Low 25(OH)D concentrations in the maternal and cord blood were significantly associated with decreased birth weight among infants of mothers carrying the rs12512631 ‘C’ allele

14	Moon, 2017 (54)	682 pregnant women (351 placebo, 331 cholecalciferol)	Relation between GC SNPs and the response to gestational cholecalciferol supplementation	GC rs2282679 positively correlated with achieved 25(OH)D status

15	Baca, 2018 (55)	882 Black and 1796 White pregnant women	Relationship between maternal vitamin D receptor, GC, and CYP27B1 SNPs and 25(OH)D concentrations	The minor allele for rs7041 was related to increased 25(OH)D and rs4588 was associated with decreased 25(OH)D levels

16	Shao, 2017 (56)	759 pregnant women	Relationship between vitamin D pathway genes, gene–environment interactions, and vitamin D levels	Gc-1f and Gc-1s genotypes had higher plasma 25(OH)D levels compared to women with Gc-2 genotype

17	Ganz, 2018 (57)	26 third-trimester pregnant/28 lactating/21 non-pregnant and non-lactating women consuming a single amount of vitamin D	Metabolic effects of GC rs7041 SNP on vitamin D biomarkers	Increased VDBP concentrations were observed only in pregnant women with GG or GT genotypes

18	Park, 2016 (58)	26 healthy pregnant/28 lactating/21 non-pregnant and non-lactating women consuming a single amount of vitamin D	The impact of the reproductive state on vitamin D biomarkers	Higher VDBP concentrations were observed in healthy pregnant women compared to non-pregnant controls

19	Doneray, 2018 (59)	30 mother-neonate pairs with serum 25(OH)D < 10 ng/ml/30 mother–neonate pairs with serum 25(OH)D > 20 ng/ml	Relationship between serum 25(OH)D and VDBP levels in mother-neonate pairs	The maternal and neonatal vitamin D concentrations were negatively correlated with their VDBP concentrations

20	Franasiak, 2017 (60)	39 infertile premenopausal women/29 regularly cycling fertile controls	Differences in VDBP concentrations between fertile and infertile women	VDBP concentrations were lower in the infertile group, compared to controls

21	Karras, 2018 (43)	70 pairs of newly delivered neonates and their mothers	Relationship between vitamin D, VDBP, and the adipokines, adiponectin, and irisin in mothers and neonates at birth and their effects on neonate anthropometric outcomes	Independent positive correlation of maternal VDBP levels with adiponectin and irisin concentrations. Strong association of VDBP and adiponectin but not irisin was found in neonates

*VDBP, Vitamin D-binding protein; T1D, type 1 diabetes; GDM, gestational diabetes mellitus; SNP, single nucleotide polymorphism; PE, preeclamsia; EOPE, early onset preeclampsia; LOPE, late onset preeclampsia; Gc, group-specific component; BP, blood pressure; FGR, fetal growth restriction; CVF, cervicovaginal fluid; PROM, preterm premature rupture of the fetal membranes; 25(OH)D, 25-hydroxyvitaminn D*.

## Critical Appraisal of Available Evidence and Reasons for Discrepancies Between Study Results

Available evidence in the field manifest several limitations, with the most prominent being the wide heterogeneity between study design, included populations, explored outcomes, and analytical methods. Therefore, any interpretation of studies results should be made with caution. Regarding genetic studies in particular, they often present specific methodological issues, including inadequate power to reveal potential gene-disease associations, population stratification as a result of genetic and environmental heterogeneity between studied populations, departure from Hardy–Weinberg equilibrium, hence, they tend to produce inconclusive and conflicting results (65).

Ethnic differences in VDBP polymorphisms could also result in differences in 25(OH)D status in pregnant cohorts across the same geographical region (66, 67), as well as the gap between observational and supplementation studies (68, 69). We have previously described in detail (69) the main reasons behind the aforementioned gap between observational and interventional studies, with regard to the role of Vitamin D in pregnancy. These reasons can be summarized as follows: 1. various study designs (lack of a precise outcome in conjunction with timing of supplementation, enrollment of participants with varied vitamin D status); 2. difficulties in the interpretation of vitamin D equilibrium (lack of determination of plasma half-life); 3. administration of a wide range of regimens, in terms of dose, bolus, and form; 4. geographical dissimilarities (vitamin D needs could vary significantly within a country, particularly in areas with a wide range of latitude gradient); 5. alterations of vitamin D metabolism during pregnancy and 6. supplementation of individuals with low baseline 25(OH)D concentrations would be more likely to have beneficial effects compared to subjects with higher baseline status. It is highly likely that the above handicaps also affect the reproducibility of study results related to VDBP status during pregnancy, since Vitamin D and VDBP are parts of a common biological network with complex interactions between its various components.

In addition, laboratory assessment of VDBP concentrations during pregnancy may be challenging. Different analytical methods have been developed and used in the conducted studies, so far. The monoclonal immunoassay technique recognizes an epitope near the polymorphic region of VDBP and thus has different affinities for the different VDBP haplotypes: this issue probably affects the results of the assay. As a consequence, it presented uncoupling results compared to a polyclonal immunoassay method (70). Hoofnagle et al. developed a liquid chromatography–tandem mass spectrometric (LC-MS/MS) assay, where plasma proteins can be cleaved into peptides making their specific detection and quantification possible (71). LC-MS/MS method gave results similar to the polyclonal immunoassay, but different from those of the monoclonal immunoassay (70, 71). In addition, although the existence of various vitamin D forms (such as epimers) has been established, their clinical significance remains obscure. Furthermore, recent data show that at least one epimer form has activity *in vitro* (72, 73). With the development of more advanced assays, a thorough understanding of the interplay among the various vitamin D forms could be achieved.

## GAPS in Existing Knowledge and Future Research Agenda

It becomes evident from the above that VDBP plays some role in the progression of normal pregnancy and that it is also implicated in the pathogenesis of some of the commonest pregnancy complications, in a way—however—that is not yet completely understood. The fact that VDBP seems to be involved in the pathogenesis of numerous and heterogeneous clinical entities, underlines its pluralistic role in vitamin D homeostasis.

Despite the intensive research work having been conducted during the past few years in terms of the role of Vitamin D in pregnancy, it is clear that existing data regarding VDBP is still very limited. The understanding of the physiology of the VDBP network is extremely useful; however, focus of future research on the association between VDBP and adverse pregnancy outcomes may have multiple benefits. First, the establishment of a novel biomarker for the early detection of endangered pregnancies, which can be translated into daily clinical benefit and second, the further decryption of the complex pathophysiological aspects of pregnancy’s abnormalities.

For this purpose, additional clinical trials are required, characterized by interventional and randomized design in order to reduce potential bias, adequate power, and targeted on populations with high-risk for adverse outcomes. Future mechanistic studies from different ethnic groups are needed to investigate the regulatory and immune functions of VDBP during pregnancy and other reproduction outcomes.

## Author Contributions

SK and TK drafted the first edition of the paper. All authors drafted the final version and contributed to the final revisions. Last revision was made by SK.

## Conflict of Interest Statement

The authors declare that the research was conducted in the absence of any commercial or financial relationships that could be construed as a potential conflict of interest.
